# Stretchable, self-healing, transient macromolecular elastomeric gel for wearable electronics

**DOI:** 10.1038/s41378-019-0047-4

**Published:** 2019-03-11

**Authors:** Mingming Hao, Lianhui Li, Shuqi Wang, Fuqin Sun, Yuanyuan Bai, Zhiguang Cao, Chunyan Qu, Ting Zhang

**Affiliations:** 1 i-Lab, Key Laboratory of multifunctional nanomaterials and smart systems, Suzhou Institute of Nano-tech and Nano-bionics, Chinese Academy of Sciences, 215123 Suzhou, China; 2 Nano Science and Technology Institute, University of Science and Technology of China, 96 Jinzhai Road, 230026 Hefei, Anhui China

**Keywords:** Materials science, Nanoscale materials

## Abstract

Flexible and stretchable electronics are emerging in mainstream technologies and represent promising directions for future lifestyles. Multifunctional stretchable materials with a self-healing ability to resist mechanical damage are highly desirable but remain challenging to create. Here, we report a stretchable macromolecular elastomeric gel with the unique abilities of not only self-healing but also transient properties at room temperature. By inserting small molecule glycerol into hydroxyethylcellulose (HEC), forming a glycerol/hydroxyethylcellulose (GHEC) macromolecular elastomeric gel, dynamic hydrogen bonds occur between the HEC chain and the guest small glycerol molecules, which endows the GHEC with an excellent stretchability (304%) and a self-healing ability under ambient conditions. Additionally, the GHEC elastomeric gel is completely water-soluble, and its degradation rate can be tuned by adjusting the HEC molecular weight and the ratio of the HEC to glycerol. We demonstrate several flexible and stretchable electronics devices, such as self-healing conductors, transient transistors, and electronic skins for robots based on the GHEC elastomeric gel to illustrate its multiple functions.

## Wearable electronics: self-healing elastomer

A macromolecular elastomeric gel offers stretchability, self-healing and controllable degradation, suggesting its potential for flexible electronic devices. Wearable electronics are of major interest for personal and smart health monitoring. A major challenge they face however is how to both resist and heal damage as a result of continuous stretching in service. Now, a team led by Ting Zhang from Suzhou Institute of Nano-tech and Nano-bionics, Chinese Academy of Sciences, demonstrates a macromolecular elastomeric gel, in which hydrogen bonding between small molecule glycerol and hydroxyethylcellulose imparts the abilities of stretching, self-healing and controllable degradation in the resulting elastomer. The authors demonstrate the potential of their material as self-healing conductors, transient transistors and as electronic skin for robots.

## Introduction

Flexible and stretchable electronics are used extensively in human-activity monitoring^1–5^, personal healthcare^3,6–9^, and human−computer interactions^10–13^, since their unique characteristics, such as having a low modulus, being lightweight, and having a high flexibility and stretchability, allow for the conformal integration of electronics with nonplanar surfaces^3,14^. However, such flexible and stretchable electronics are still susceptible to tearing, puncturing and other forms of mechanical failure, leading to a loss in electrical performance, which greatly limits their further application. Encapsulation is an effective way to protect these electronics, but sacrifices its stretchability, lightness, and thickness. Endowing flexible and stretchable electronics with a self-healing ability to resist mechanical damage is insistently demanded for future flexible and stretchable electronics^15^. Moreover, with the rapid development of flexible and stretchable electronics, the prevention of sensitive data leakage, the reduction of electronic waste, and biomedical implants without a second surgery are gradually becoming new requirements for their applications. Transient electronic devices that have the ability to disappear in a controlled manner are also extremely desirable.

In recent years, remarkable advances in self-healing materials^16–20^ and transient electronics^21–26^ have been made. For example, a healable thermoplastic elastomer material based on the use of supramolecular interactions for spontaneous healing was reported by Leibler, which can structurally heal after more than 1 week at 23 °C ^20^. Recently, Bao and colleagues demonstrated an intrinsically self-healable composite conductor composed of a supramolecular polymer (made from dimer acid, triamine and urea) and nanostructured nickel microparticles. The composite showed a good self-healing ability and stretchability (approximately 35%)^17^. In the field of transient electronics, Yu and coworkers reported a moisture-triggered physically transient electronic that was composed of functional devices on top of hydrolytically liable polyanhydride substrates. However, it takes dozens of hours for the transient electronics to degrade^24^. To the best of our knowledge, despite their rapid development, flexible electronics based on multifunctional materials that are simultaneously stretchable, self-healing and have transient properties have not yet been reported.

**Fig. 1:**
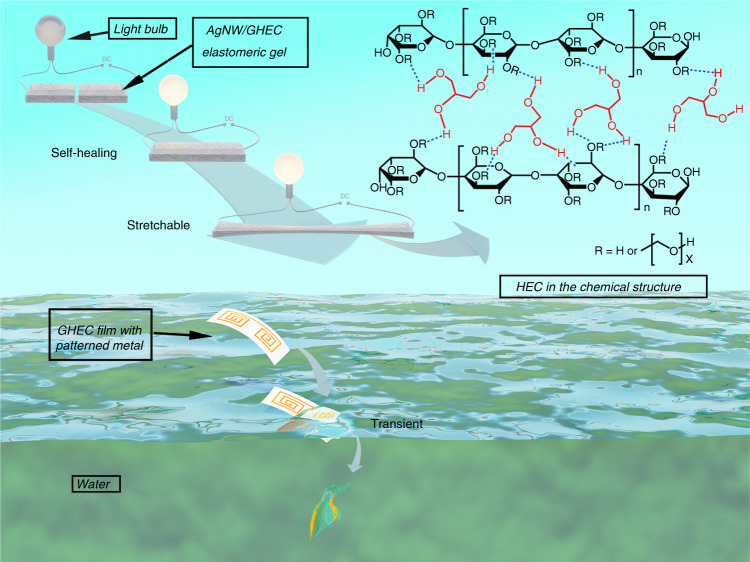
**Schematic of the GHEC elastomeric gel that can self-heal, stretch, and degrade**

Here we report a unique macromolecular elastomeric gel with the abilities of stretchability, self-healing properties and transient properties at room temperature (Fig. 1). The multifunctional elastomeric gel is fabricated by pouring an aqueous solution of a glycerol and hydroxyethylcellulose mixture. Folded hydroxyethylcellulose (HEC) molecule chains and the reversible formation of the macromolecular and the small molecule hydrogen-bonding networks are introduced by the abundant hydroxy groups, providing the GHEC with an excellent stretchability and self-healing function. The GHECs show a superior stretchability (over 304% strain (*ε*)) and a fast moisture-triggered self-healing time (~11 min) at room temperature (25 °C) and ambient humidity (40% RH). Additionally, the GHEC also exhibits a controllable transient behavior, which is attributed to the solubility of GHEC in water. By varying the ratio of glycerol to HEC in the GHEC, the desired time scales for the transience range from minutes to hours. Moreover, a series of flexible and stretchable electronics devices, such as self-healing conductors, transient transistors, and electronic skins for robots, are designed and fabricated to demonstrate the aforementioned multiple functions.

## Materials and methods

### Materials

Hydroxyethylcellulose (HEC, 200, 2000, and 5000 mPa.s, 25 °C) was purchased from Shanghai Aladdin Industrial Corporation Co. Ltd. Glycerol (analytical grade) was purchased from Shanghai Sinopharm Chemical Reagent Co. Ltd. Silver nanowires (AgNWs) (0.05 wt% purity, diameter: 15−20 nm, and length: 30−40 μm) and single-walled carbon nanotubes (SWCNTs)(>98 wt%, diameter: 5−8 nm, and length: 0.5−5 μm) were purchased from Nanjing XFNANO Materials Tech Co. Ltd.

### Synthesis and fabrication of GHEC elastomeric gel film

HEC (2 g) was added to deionized water (DI water 98 mL) at 25 °C. After stirring for 2 h, glycerol (0, 1, 2, 5, 10, and 20 g) was added dropwise and stirred continuously for 1 h. Then the mixture solution was stirred for 6 h at 80 °C and cooled to a set temperature, forming a GHEC aqueous solution. The typical procedure for the preparation of GHEC films is as follows: The mixture solution (20 mL) was poured into a quartz tank mold measuring 5 cm length × 3 cm width × 4 cm height and was dried at 80 °C for 12 h. The polymer films were then peeled off from the quartz tank mold for further testing.

### Preparation of the self-healing electrode

5 mL 0.05 wt% AgNWs dispersion were dispersed into the 100 mL GHEC aqueous solution by stirring for 4 h. Then the mixture was poured into a quartz tank mold forming a self-healing AgNW/ GHEC elastomeric gel.

### Preparation of transient electronics

The transient electronics were fabricated by patterning metal aluminum (Al) electrodes onto the GHEC films using electron beam evaporation via a shadow mask. A piece of GHEC film was first rinsed by sonication in ethanol for 10 min and then was blow-dried with a nitrogen gun to obtain a piece of clean degradable substrate. Then the film was treated using UV ozone for 15 min. Then the electrode was assembled using electron beam evaporation at 200 °C for 5 min at a rate of 0.8−1.2 nm s^−1^.

### Preparation of multifunctional electronic skins

The multifunctional electronic skins were fabricated by assembling a stress-sensitive layer of SWCNTs on a GHEC film. Initially the SWCNT suspension (ink) was fabricated: 2 mg of SWCNTs (>98 wt%, with an average diameter between 5 and 8 nm, and average length between 0.5 and 5 μm Nanjing XFNANO Materials Tech Co., Ltd., China) were dispersed in a 100 mL absolute ethyl alcohol by strong sonication (400 W) for 2 h using an ultrasonic cell disruptor (BILON 92-IIShanghai Bilon Co., Ltd). Then the SWCNT suspension (ink) was spray-coated onto the GHEC film. Finally, the coated film was dried at 80 °C for 2 h, forming a multifunctional electronic skin.

### Materials characterization and the sensor experiments

A digital camera (Canon EOS 70D) was used to take photos and record videos. Tensile tests were performed using the Instron 3365 at a constant speed of 10 mm min^−1^.

#### Dynamic mechanical analysis (DMA)

The storage moduli and loss moduli of the elastomeric gel samples (film, 10 mm in length, 5 mm in width and 0.4 mm in thickness) were measured using a NETASCH DMA 242 E with a compression mode at 10 Hz in the temperature range of 25−105 °C with a heating rate of 5 °C min^−1^, and an amplitude of 100 μm. The measurement was performed in normal air atmosphere. The FT-IR spectra were collected via a Thermo Nicolet iN 10 spectrometer from 4000 to 400 cm^−1^ at 77 K in a liquid nitrogen cooling environment. SEM was performed for micromorphology observation using a Hitachi S-4800 cold field emission scanning electron microscope at an accelerating voltage of 5 kV. The current measurements were performed by connecting two ends of the AgNW/GHEC film with a digital source meter (Keithley 2602 A), using two copper wires as the electrodes to record the real-time electric current (*I*) of the film under a constant voltage (*V*_0_) of 2 V.

## Results and discussion

HEC is an important and abundant cellulose derivative with advantages of good biocompatibility and biodegradation properties^27–29^, which make it a potential candidate for multifunctional flexible and stretchable transient electronics devices. However, the bulky HEC molecular chains fold and interweave with one another due to the intermolecular forces (Van Der Waals force, including the hydrogen bond), resulting in a strong steric hindrance, limited stretchability, and approximately no self-healing ability^30^. To meet the requirements of multifunctional flexible and stretchable transient electronics devices with a self-healing ability, a series of supramolecular GHEC elastomeric gels were synthesized by introducing different amounts of functional guest small glycerol molecules into the HEC matrix to form new elastomeric oil gels. GHEC elastomeric gel is stable and the weight changes of the GHEC gel are less than 0.5% under different temperatures (0~90 °C) at both 20 and 80% RH (Figure S1).

**Fig. 2:**
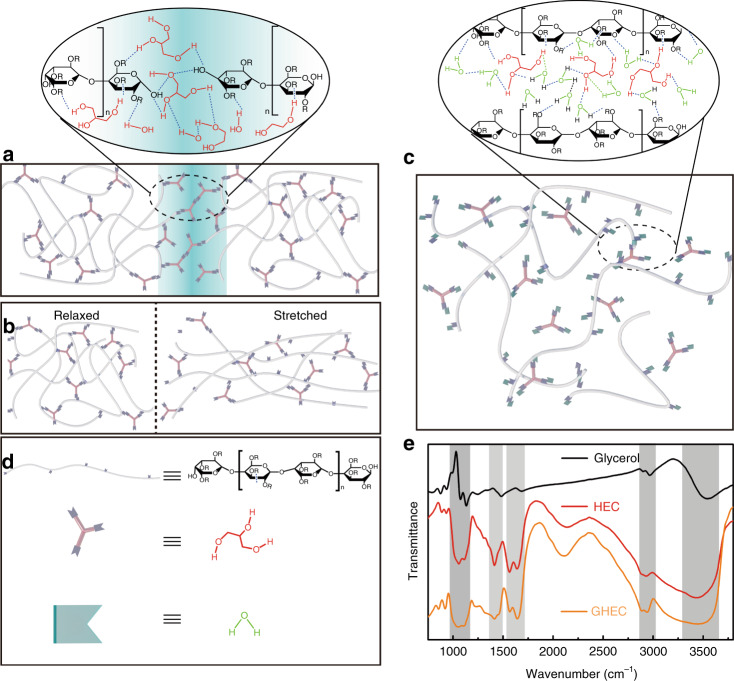
Working mechanism of the GHEC elastomeric gel. **a**–**d** Schematic diagrams of the self-healing, stretching, and degrading properties of the GHEC elastomeric gel. Gray lines represent the polymer chains of HEC and form cross networks. Purple shapes on the polymer chains and brown short lines form the primary hydrogen bonds between the polymer chains and glycerol. The blue-green shapes represent water molecules. **e** The Fourier transform infrared spectra of various materials; the orange, black, and red lines represent glycerol, HEC and GHEC, respectively. HEC, hydroxyethylcellulose; GHEC glycerol/hydroxyethylcellulose

Since hydrogen bonds are weak dynamic noncovalent bonds^15,31^, they will break preferentially upon damage. However, those broken hydrogen bonds can reform at the fractured interfaces due to the dynamic characteristic of supramolecular chains (Fig. 2a), which is the self-healing mechanism of the GHEC. For the insertion of small glycerol molecules, the reformation of dynamic hydrogen bonds occurs between the HEC chains and the guest small glycerol molecules (Fig. 1), which endows the HEC supramolecular chains with mobility and makes the folded HEC molecular chains easier to straighten, inducing a higher stretchability for the GHEC compared with HEC (Fig. 2b). Additionally, the GHEC elastomeric gel is water-soluble and the fundamental mechanism of dissolution can be described as follows: first, water molecules interact with the glycerol molecules at the outer layer of the GHEC. Then, the glycerol molecules between the chains of the HEC molecules are replaced by water molecules and the glycerol dissolves, which promotes the binding of water molecules to the HEC. The same process is conducted from the outer layer to the inner layer, and the GHEC is finally completely dissolved^32,33^. Therefore, its degradation rate can be tuned by adjusting the HEC molecular weight or the ratio of HEC to glycerol (Fig. 2c). To verify the molecular composition of the GHEC, the infrared spectra of the GHEC was measured. As Fig. 2e presents, the Fourier transform infrared (FTIR) spectra of the GHEC includes peaks belonging to O−H stretching at 3500 cm^−1^, C−H stretching at approximately 2800−3000 cm^−1^ and C−O−C stretching at approximately 1100 cm^−1^, which are the characteristic peaks of HEC (O−H, C−H, C−O−C) and glycerol (O−H, C−H), respectively. FTIR spectra suggests that the HEC and glycerol are simply physically mixed in the GHEC instead of cross-linking by chemical reactions. Hence, the hydroxyl functional group is not destroyed, which supports the aforementioned mechanism.

### Mechanical properties of GHEC elastomeric gels

**Fig. 3:**
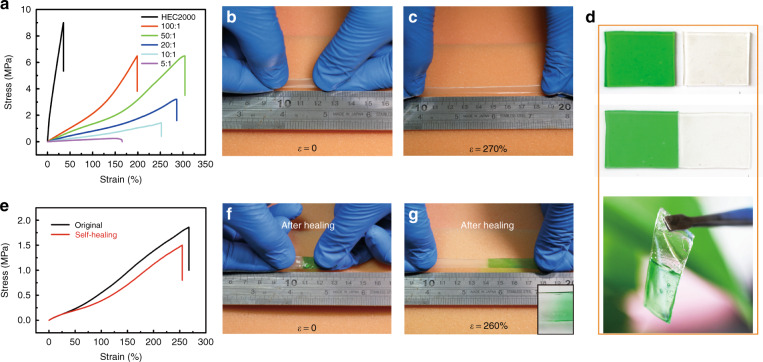
Mechanical and self-healing properties characterization. **a** Stress−strain curves of the elastomeric gel films prepared with different mass ratios of glycerol to HEC2000 stretched at a constant speed of 10 mm/min for samples of 10 mm length, 5 mm width and 0.4 mm thickness. **b**, **c** Optical images of the GHEC (*m* = 20) elastomeric gel film before and after stretching and showing high stretchability at 270% strain. **d** Optical images of the healing process of two GHEC (*m* = 20) elastomeric gel films (top). The films were put together at room temperature (25 °C) and 50% ambient humidity (bottom). **e** Stress−strain curves of the original and healed GHEC elastomeric gel films that are shown in (**d**). **f**, **g** Optical images of the GHEC after self-healing under various strains. GHEC glycerol/hydroxyethylcellulose

The mechanical properties of a series of GHEC elastomeric gels were characterized. As shown in Fig. 3a, the typical tensile stress−strain curves of the GHECs elastomeric gel films with various mass ratios (*m*) of the 2% HEC2000 aqueous solution (the viscosity of HEC is 2000 mPa.s) to glycerol display a decreased elasticity modulus from 25.78 to 0.02 MPa as *m* decreased from 1 to 5:1. However, the stretchability of GHEC with *m* = 50 of 304% is the best, and decreased when *m* was less than 50 since the numerous glycerol molecules form more weak hydrogen bonds between the glycerol molecules, which preferentially break before the HEC chains completely spread out^16,32,34^. In particular, the elasticity modulus of the GHEC with *m* = 20 is approximately 0.91 MPa and the max strain is 270%, which are soft enough for wearable flexible and stretchable electronics (Fig. 3b, c). In addition to *m*, the HEC molecular polymerization degree is another key factor that affects the mechanical properties of the GHEC elastomeric gel. As shown in Figure S2a and b, the GHEC films with larger viscosity HEC molecules exhibit stronger elasticity moduli and worse stretchability, which are attributed to the larger molecule chains inducing a greater steric hindrance.

### Characterization of the self-healing ability of the GHEC

For the presence of dynamic hydrogen bonds in the GHEC elastomeric gel, the reversible formation of the hydrogen bonds provides the GHEC with a preeminent self-healing ability, which is proven by the healing process shown in Fig. 3d. The mechanical properties are one of the most effective ways to evaluate the material’s healing efficiency, because they take the restoration of both the stress and strain into account. We define the mechanical healing efficiency *η*_m_ as the ratio of the restored maximum strain to the original maximum strain. To investigate the healing performance of GHEC2000, the stress−strain curves of a GHEC2000 film before and after healing were measured. As presented in Fig. 3e, after healing for 8 min in ambient humidity (50% RH) and temperature (25 °C), *η*_m_ reaches up to 96.3%, which is further demonstrated by the optical pictures (Fig. 3f−g, Movie 1). The interface of the two fractures (the green and colorless parts) after healing is hazy, as shown in the inset of Fig. 3g, proving that the healing process is a typical supramolecular assembly with dynamic hydrogen bonds.

**Fig. 4:**
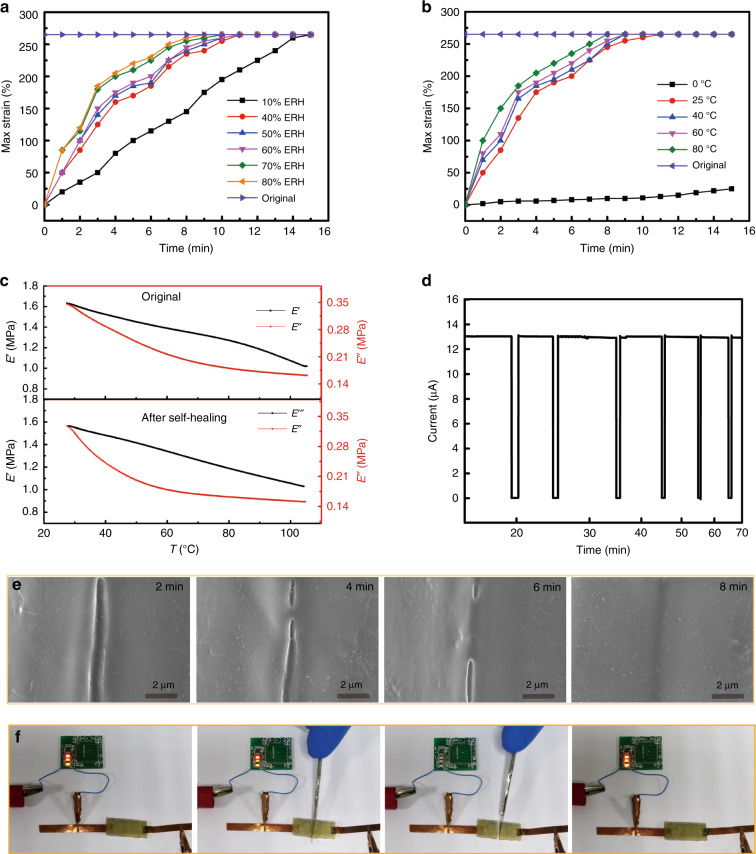
The main influencing factors and the electricity self-healing property characterization. **a** The maximum strain−time curves of the GHEC films healed under various ambient humidities at constant room temperature (25 °C). **b** The maximum strain−time curves of the GHEC films healed at various ambient temperatures under a constant ambient humidity (50%). **c** The dynamic mechanical analysis results of the GHEC (*m* = 20) elastomeric gel films. Variations of the storage moduli and loss moduli before and after self-healing as a function of the temperature from 25 to 105 °C. **d** Repeated electrical healing for six cuts at the same severed location by the current−time curve at room temperature. **e** SEM microscope images showing the self-healing process of AgNW/GHEC. **f** Optical images of a battery-powered circuit to further prove the potential of electromechanical self-healing for electronic circuits. GHEC glycerol/hydroxyethylcellulose

To further understand the self-healing properties of the GHEC elastomeric gel system, the self-healing processes under various environmental conditions were investigated. Figure 4a depicts the maximum strain of the GHEC2000 elastomeric gel films with *m* = 20 during the self-healing process under various ambient humidities from 10 to 80% RH at a constant temperature of 25 °C. As illustrated, it only takes 9 min to achieve 100% mechanical repair under 80% RH and 15 min to achieve the same healing level under 10% RH, which confirms that the restoration behavior of the GHEC has no obvious requirement for humidity but that a higher humidity can effectively accelerate the self-healing speed. These results can be interpreted as being due to the water molecules improving the HEC chain’s dynamic and assembly activities, thereby quickening the healing process. A kinetically increasing temperature can also increase the dynamic activity of the molecule chains, and thus the influence of temperature on the self-healing properties of the GHEC deserves to be studied. As seen from Fig. 4b, the healing speed of the GHEC2000 elastomeric gel films indeed increases as the ambient temperature increases from 0 to 80 °C when the ambient humidity was stable at 50% RH. Interestingly, the healing time required to achieve a 100% restoration efficiency at room temperature, 25 °C, is only 11 min. However, 15 min is required to attain 10% *η*_m_. The self-healing speed plummets when the temperature reaches 0 °C, which is attributed to the water molecules crystallizing from the liquid to the solid state, which greatly hinders the molecule chain dynamics and the assembly activities, thus severely limiting the repair speed. Moreover, a dynamic mechanical analysis (DMA) was conducted to inspect the dependence of the moduli of the GHEC (both before and after self-healing) on the temperature because the mechanical properties of the GHEC elastomeric gel can greatly affect the performance of flexible wearable electronics. Figure 4c shows the variation in the storage moduli (*E*′) and loss moduli (*E*″) of the representative GHEC elastomeric gel (up: original; down: after self-healing) as a function of the temperature over a range from 30 to 105 °C. Apparently both the *E*′ and *E*″ values of the GHEC elastomeric gel before and after self-healing drop rapidly with an increase in the temperature from 30 to 105 °C, which suggests that the GHEC elastomeric gel becomes weak and softens with an increase in the temperature due to the dynamic breakage of intermolecular hydrogen bonds between the glycerol and HEC. Additionally, the DMA curves of the GHEC elastomeric gel before and after self-healing have no obvious attenuation, demonstrating the excellent mechanical self-healing ability.

In addition to the mechanical properties, the electrical property restoration is another valuable way to characterize the self-healing ability of the GHEC. Figure S3 shows an SEM image of an AgNW/GHEC elastomeric gel film produced by casting a uniformly dispersed silver nanowire (AgNWs) GHEC aqueous solution. Such an AgNW/GHEC elastomeric gel displays a good electrical conductivity due to the formation of AgNWs networks in the elastomeric gel matrix. To investigate the electrical healing, the AgNW/GHEC elastomeric gel film was completely bifurcated using a scalpel and the two fractured surfaces were brought together. A digital source meter (Keithley 2602A) was used to record the real-time current with a constant applied voltage of 1 V DC. As illustrated in Fig. 4d, once bifurcated the current immediately goes to 0, forming an open circuit. As the edges of the elastomeric gel were brought into contact, the conductivity increased, and the current returned to the initial value within 1 s. The elastomeric gel became self-supporting after 30 s, demonstrating the excellent repeatable restoration of the electrical performance. The series of SEM images in Fig. 4e exhibits details of the AgNW/GHEC elastomeric gel film healing process. Clearly the AgNWs at the edges of the elastomeric gel can immediately contact one another, forming new conductive networks, which results in the fast restoration of the electrical performance. Subsequently, supermolecular chains diffuse, cross-link with one another, gradually begin to be self-supporting, and eventually achieve complete mechanical properties restoration. Finally, a battery-powered circuit was constructed to further prove the potential of electromechanical self-healing for electronic circuits (Fig. 4f, Movie 2). The LED light in the series circuit are extinguished once cut off from the self-healing conductive elastomeric gel and then were lit again after transitory healing. Electrical and mechanical performances during ten instances of failures and recoveries (cutting-self-healing cycles) were characterized to further demonstrate the stability. As shown in Figure S4, the maximum strain of AgNW/GHEC decreases 5% and the resistance increases 0.46% after ten instances of failures and recoveries, verifying the repeatable restoration of the electrical and mechanical performances.

### Degradability characterization of GHEC elastomeric gel

**Fig. 5:**
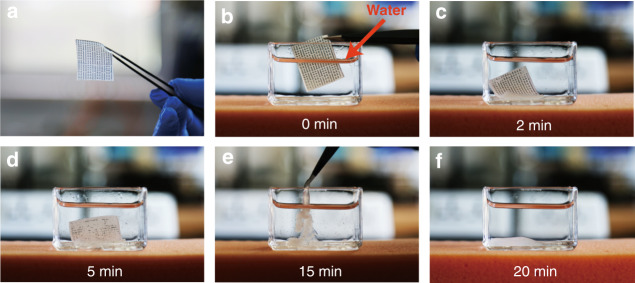
transience process of the GHEC film with patterned metal aluminum (Al) electrodes. **a**–**f** Optical images of the transience process of the GHEC film with patterned metal aluminum (Al) electrodes during various degradation stages. GHEC glycerol/hydroxyethylcellulose

Sensitive data leakage prevention, the reduction of electronic waste, and biomedical implants without second surgery are urgent requirements for controllable transient electronics. The GHEC elastomeric gel system is water-soluble and the degradation rate can be tuned by adjusting the HEC molecular weight or the ratio of HEC to glycerol, which makes GHEC a promising transient electronic substrate. Figure 5a shows a 1-mm-thick GHEC2000 (*m* = 20) film with patterned metal aluminum (Al) electrodes that was prepared using electron beam evaporation. Optical photographs (Figure 5b–f) detail the GHEC film degradation process after immersion in 25 °C water: The GHEC substrate absorbs water and begins to swell within 2 min. Meanwhile, the electrode falls off the HEC substrate and disintegrate into small fragments, which represents the loss of structure and electrical performance of the flexible electrodes system. After 20 min of degradation, all the electrodes disappear, and the substrate becomes a fluid flocculation, which proves that GHEC can be used as a rapidly transient electronic device. To further investigate the degradation property of the GHEC, a series of GHEC with different components were fabricated. As shown in Figure S5, the degradation rate of the GHECs (*m* = 20) with a low viscosity of HEC is obviously faster than that of the GHECs with a high viscosity of HEC, which is caused by the fact that short chains have less intermolecular interaction sites^32^. Moreover, as the increase in glycerol can also effectively decrease the HEC intermolecular interaction sites, promoting the GHEC degradation rate, it therefore takes a shorter time for the GHECs with a small *m* to degrade (for example, the degradation time is approximately 60 min for GHEC with *m* = 20, while the degradation time is more than 90 min for that with *m* = 50, shown in Figure S6). The ambient temperature is also an important factor that influences the degradation rate, and a high temperature can accelerate the degradation rate of the thermal motion of molecules (Figure S7). Additionally, the electrical properties of the material do not change along with the mass ratios of glycerol and HEC or the viscosities of HEC, because GHEC is a nonionic mixture of glycerol and HEC, which has weak electrical conductivity in ambient conditions. The resistance of GHEC gel (*m* = 20) film decreases from 8 to 3 MΩ after immersion in water, which is greater than AgNW/GHEC (75 kΩ), shown in Figure S8. Assembling conductive materials (such as AgNWs, CNTs, etc.) in or on a GHEC film is the main way to endow GHEC gel with conductivity.

### Demonstration of electronic skin based on the self-healing transient stretchable GHEC elastomeric gel

**Fig. 6:**
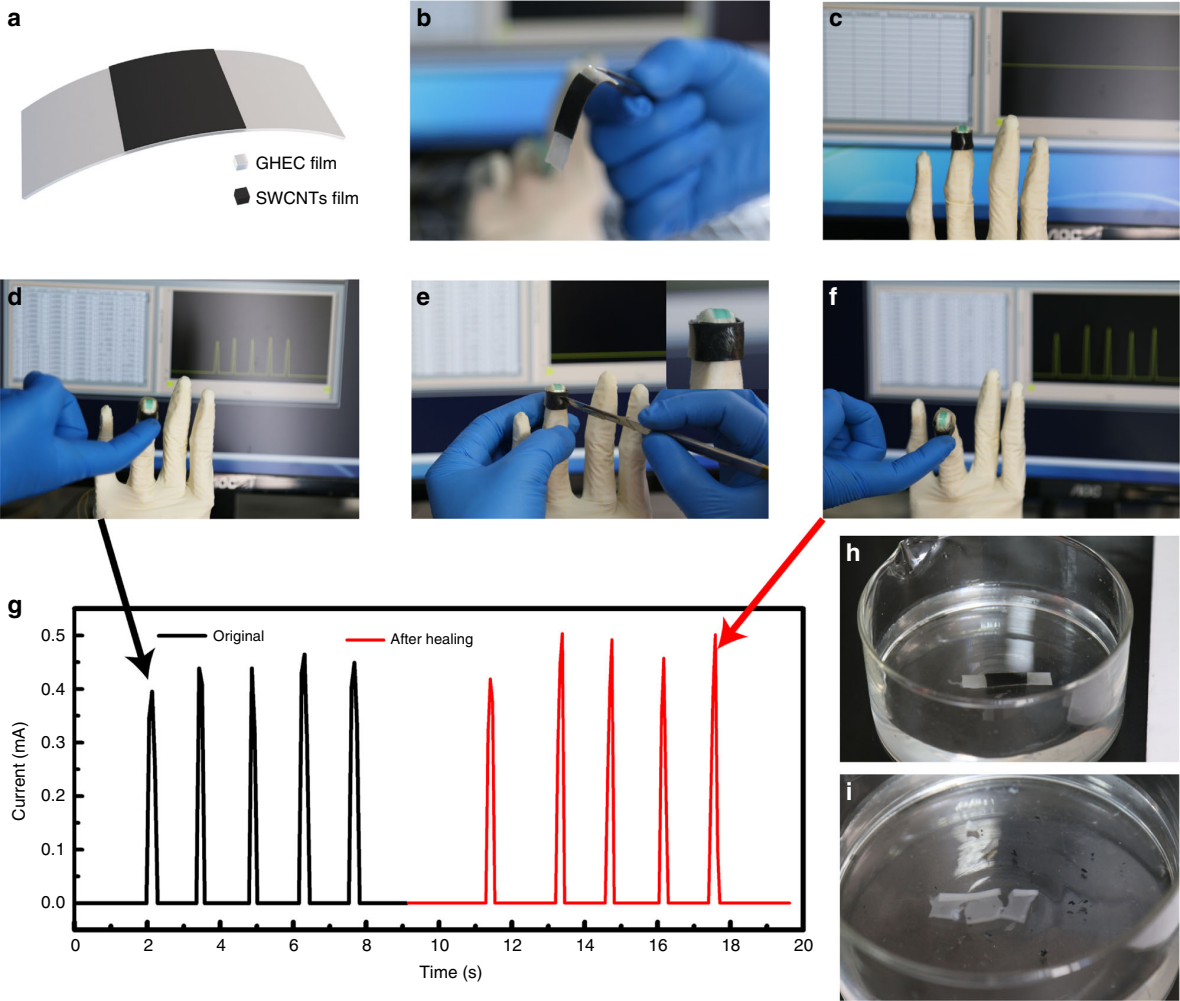
Self-healing, transient stretchable electronic skin. **a**, **b** Schematic diagram and optical image of an electronic skin, respectively. **c** Optical image showing electronic skin assembled onto a manipulator ring finger by being wrapped around the finger. Inset: optical photograph of a manipulator wearing a latex glove. **d** Optical photograph showing the process of touching a human finger. **e** Optical photograph showing the process of cutting a wound on the electronic skin using a scalpel. **f** The process of touching a human finger for the electronic skin after self-healing. **g** Current−time curve of the electronic skin during the process (**d**–**f**). **h**, **i** Optical photographs showing the degradation process of the electronic skin

Finally, a practical application of the self-healing transient stretchable GHEC was demonstrated by assembling a stress-sensitive layer of SWCNTs on a multifunctional GHEC film to form an electronic skin (Fig. 6a), which is widely used in wearable electronics and human−machine interfaces. Figure 6b shows a piece of electronic skin (see Methods for the fabrication method), which can be assembled onto the curved surface of a manipulator ring finger by being wrapped around the finger, since the cross-section of the electronic skin automatically heals under environmental conditions (Fig. 6c). Figure 6d and Movie 3 (Supporting Information) present the real-time pressure detection of the ring finger. As the finger bends, driven by the controller, to touch the object repeatedly, its current shows a periodic synchronous increase and decrease, achieving real-time pressure sensing, which is very important for creating intelligent robot perception and human−computer interaction. The real-time current of the electronic skin was measured using a digital source meter (Keithley 2602A) under a given voltage of 1 V. Moreover, to simulate a real mechanical injury, we used a scalpel to cut a wound on the electronic skin (Fig. 6e and Movie 3). After a short period for self-healing, the manipulator ring finger regains its sensing ability, which is approximately identical to its previous sensing performance, as shown in Fig. 6f, g (the slight difference between each cycle resulted from the slight discrepancy of each bend), indicating a good self-healing ability to resist mechanical damage. The stability of the electronic skin is further proven by ten cycles of repeated failures and recoveries (Figure S9). Finally, the electronic skin was removed and immersed in water for degradation (Fig. 6h, i). The multifunctional electronic skin demonstrates the abilities of stretchability, self-healing and degradation of the GHEC, which extend the flexible wearable electronic applications through the prevention of sensitive data leakage, the reduction of electronic waste, and allowing biomedical implants without a second surgery.

## Conclusions

In summary, a series of stretchable macromolecular elastomeric gels with both self-healing and degradation abilities under room temperature were developed. Based on this, a series of electronics devices were demonstrated that can work after wound healing and then degrade controllably. By inserting small molecule glycerol into HEC, forming a GHEC elastomeric gel system, dynamic hydrogen bonds occur between the HEC chain and the guest small glycerol molecules, which endows the GHEC with an excellent stretchability and a self-healing ability under normal environment conditions. Additionally, the GHEC elastomeric gel system is water-soluble and its degradation rate can be tuned by adjusting the HEC molecular weight or the ratio of HEC to glycerol. The multifunctional GHEC, which simultaneously has the abilities of self-healing to resist mechanical damage, maintains a stable performance for a desired amount of time and completely disappears under controlled programs, extends the flexible wearable electronic applications through the prevention of sensitive data leakage, the reduction of electronic waste, and allowing biomedical implants without a second surgery.

## Supplementary information

Supplemental material

Mechanical healing efficiency test

Electrical property healing efficiency test

Real-time monitoring of self-healing, transient stretchable electronic skin
