# Supplementary material for: Stretchable, self-healing, transient macromolecular elastomeric gel for wearable electronics
Source: Microsyst Nanoeng. 2019 Mar 11;5:9. doi: 10.1038/s41378-019-0047-4 (PMC6409363; doi:10.1038/s41378-019-0047-4)
Supplement: Supplementary file 1 — Supplemental material [file 41378_2019_47_MOESM1_ESM.docx]

**Title：**Stretchable, Self-healing, Transient Macromolecular Elastomeric Gel for Wearable Electronics

*Mingming Hao^+^, Lianhui Li^+^, Shuqi Wang, Fuqin Sun, Yuanyuan Bai, Zhiguang Cao, Chunyan Qu, Ting Zhang**


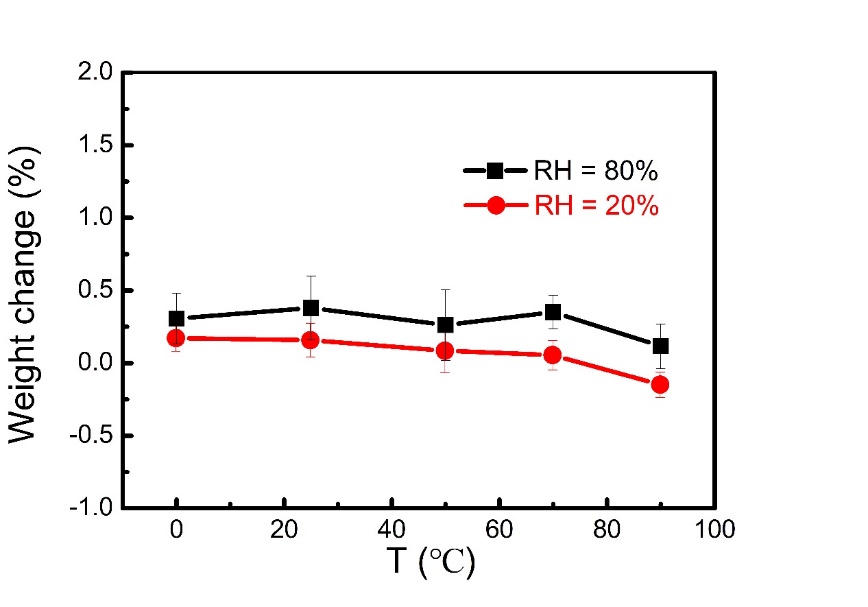


**Figure S1**. The weight change of GHEC gel (*m* = 20) under different temperature at 20% RH and 80% RH.


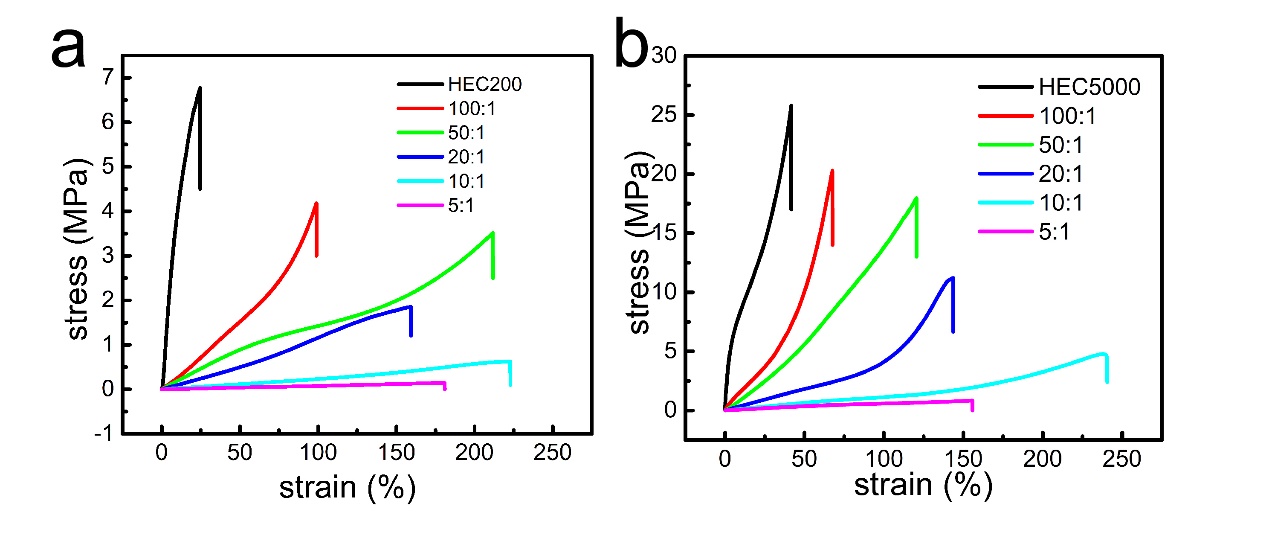


**Figure S2.** Stress-strain curves of the elastomeric gel films prepared with different mass ratios of glycerol to HEC200 (a) and HEC5000 (b) stretched at a constant speed of 10 mm/min for samples of length 10 mm, width 5 mm and thickness 0.4 mm.


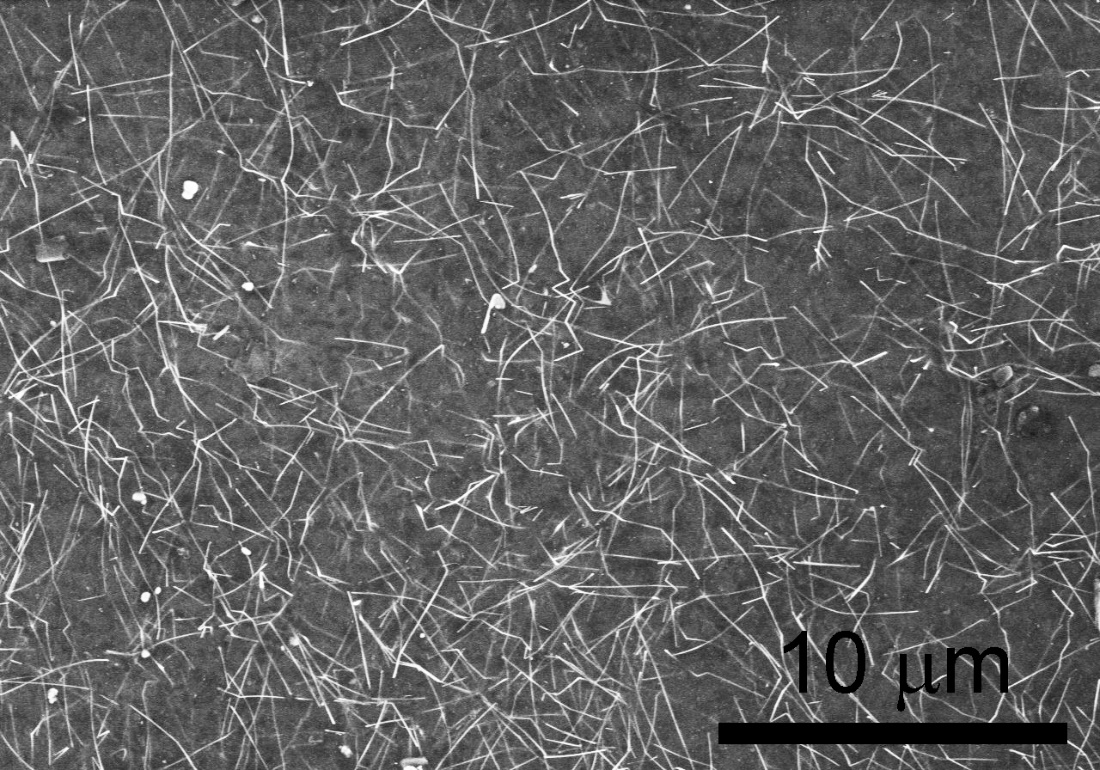


**Figure S3** SEM image of AgNW/GHEC composite film


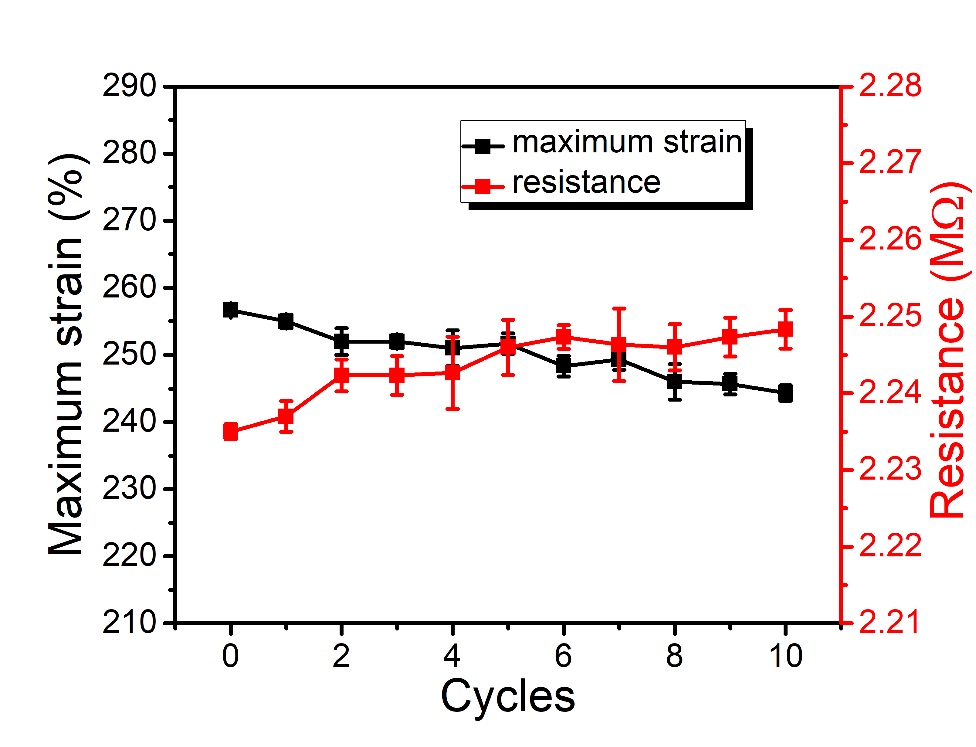


**Figure S4.** Maximum strain and resistance of GHEC gel (*m* = 20) the during 10 times failures and recoveries respectively.


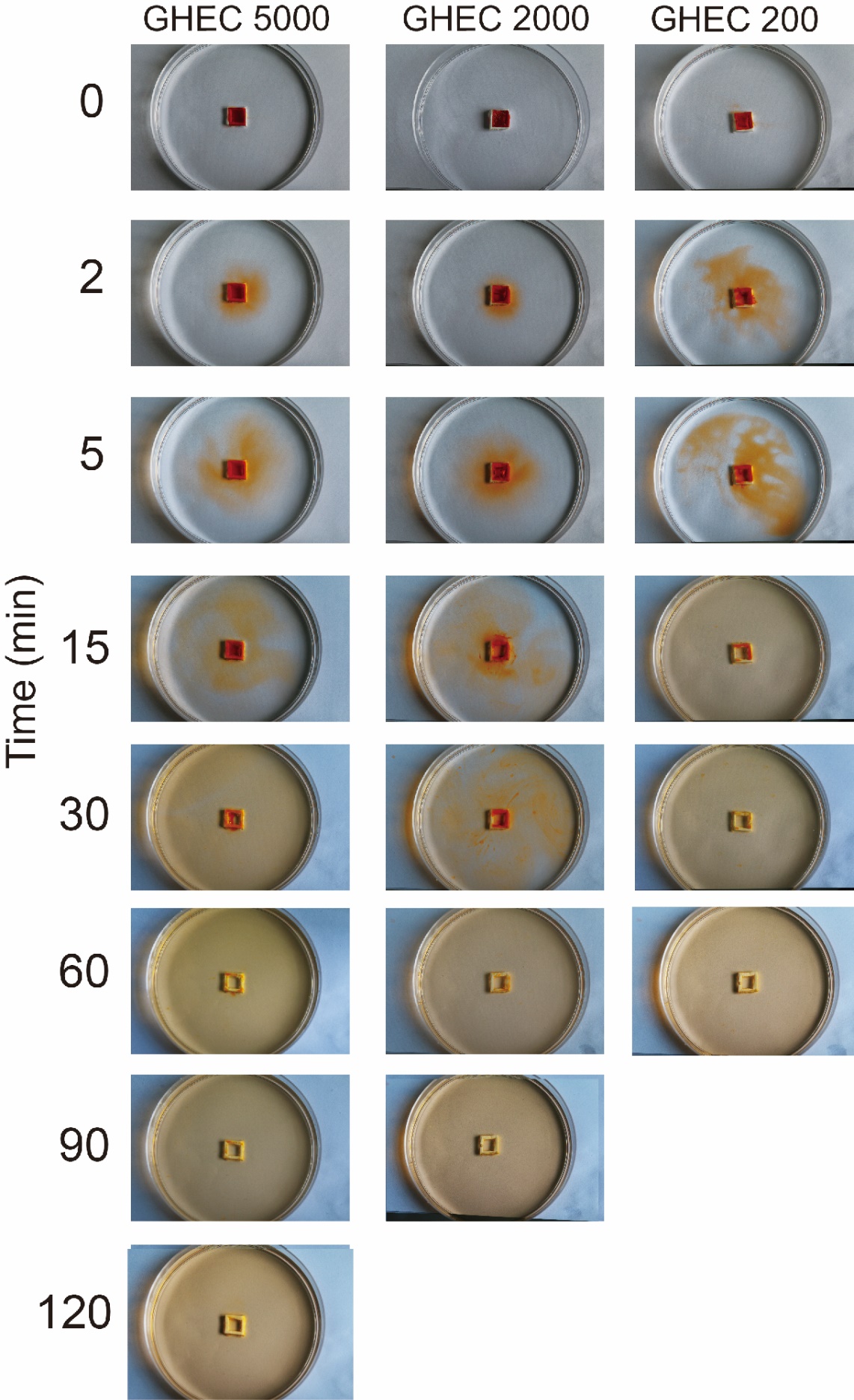


**Figure S5.** Optical images of the transience process of the GHEC elastomeric gel films with different viscosities HEC of 200, 2000, and 5000 mPa.s. The degradation process was performed by placing a 1*1 cm^2^, 150-μm-thin film on a scaffold in a culture dish with distilled water. The water temperature is 25 °C and the mass ratio (*m*) of the GHEC elastomeric gel films is 20.


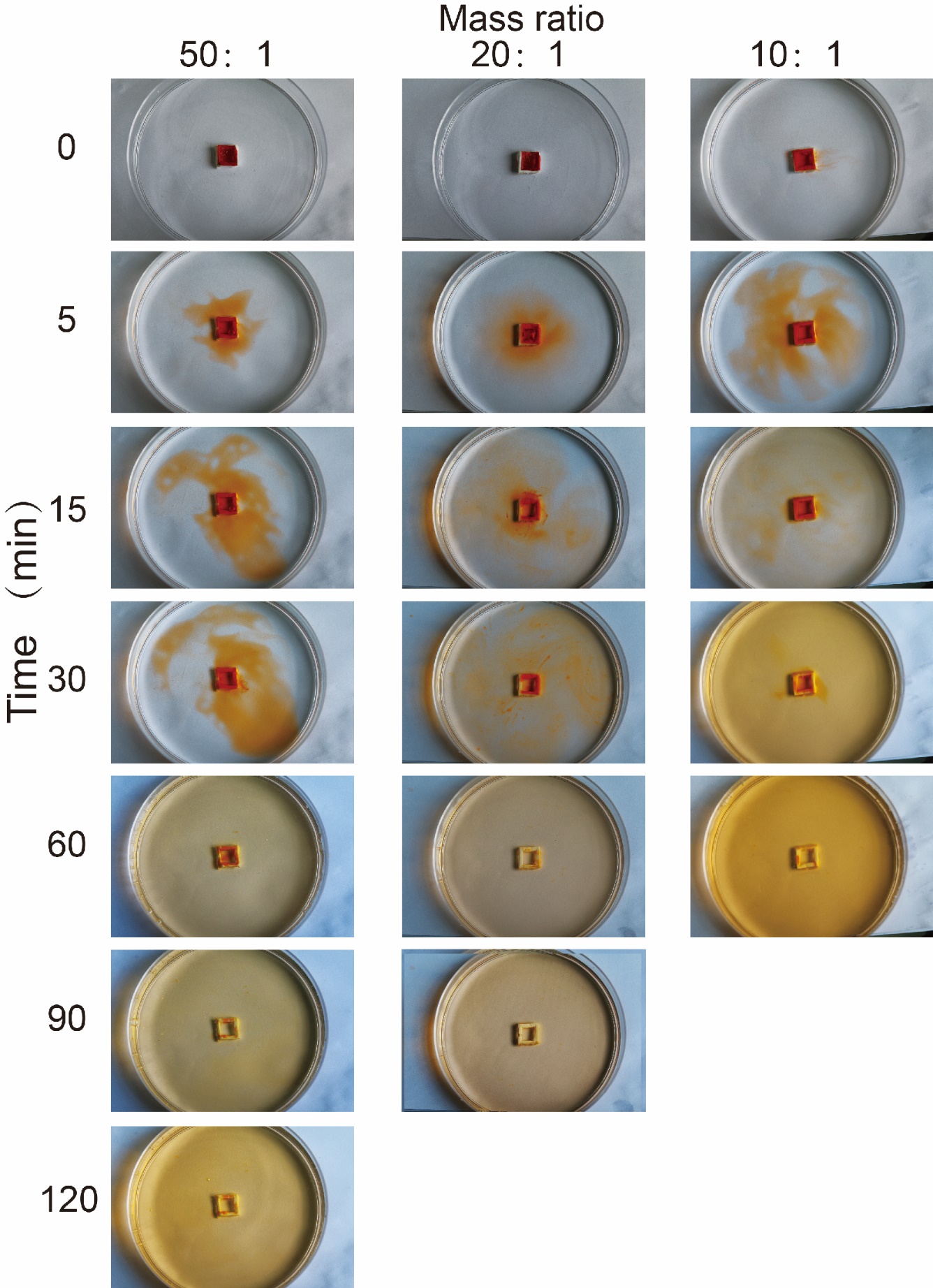


**Figure S6.** Optical images of the transience process of the GHEC2000 elastomeric gel films with different mass ratios (*m*) of 10, 20, and 50. The degradation process was performed by placing a 1*1 cm^2^, 150-μm-thin film on a scaffold in a culture dish with distilled water. The water temperature is 25 °C.


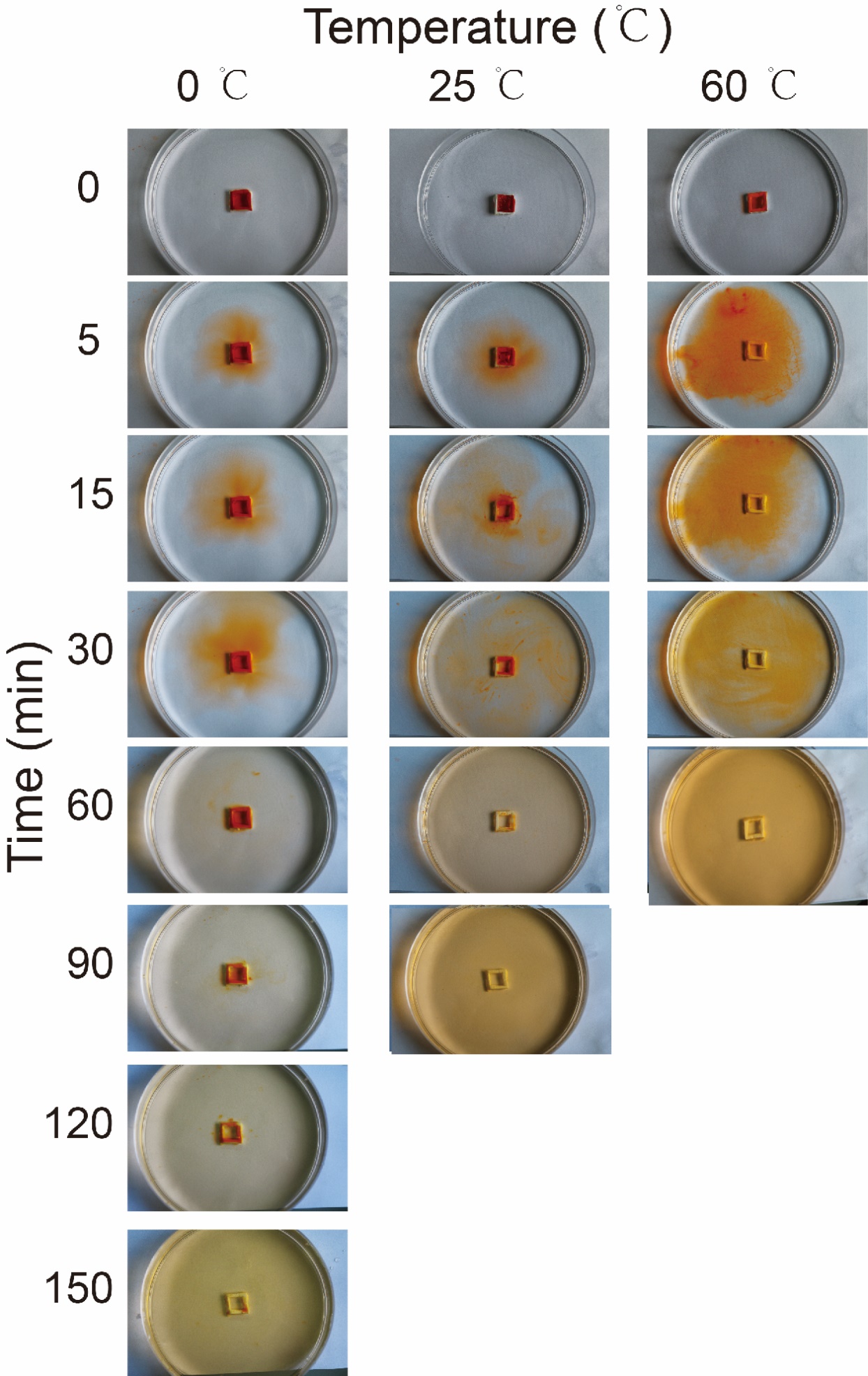


**Figure S7**. Optical images of the transience process of the GHEC2000 elastomeric gel films with *m* = 20 under different temperature. The degradation process was performed by placing a 1*1 cm^2^, 150-μm-thin film on a scaffold in a culture dish with distilled water.


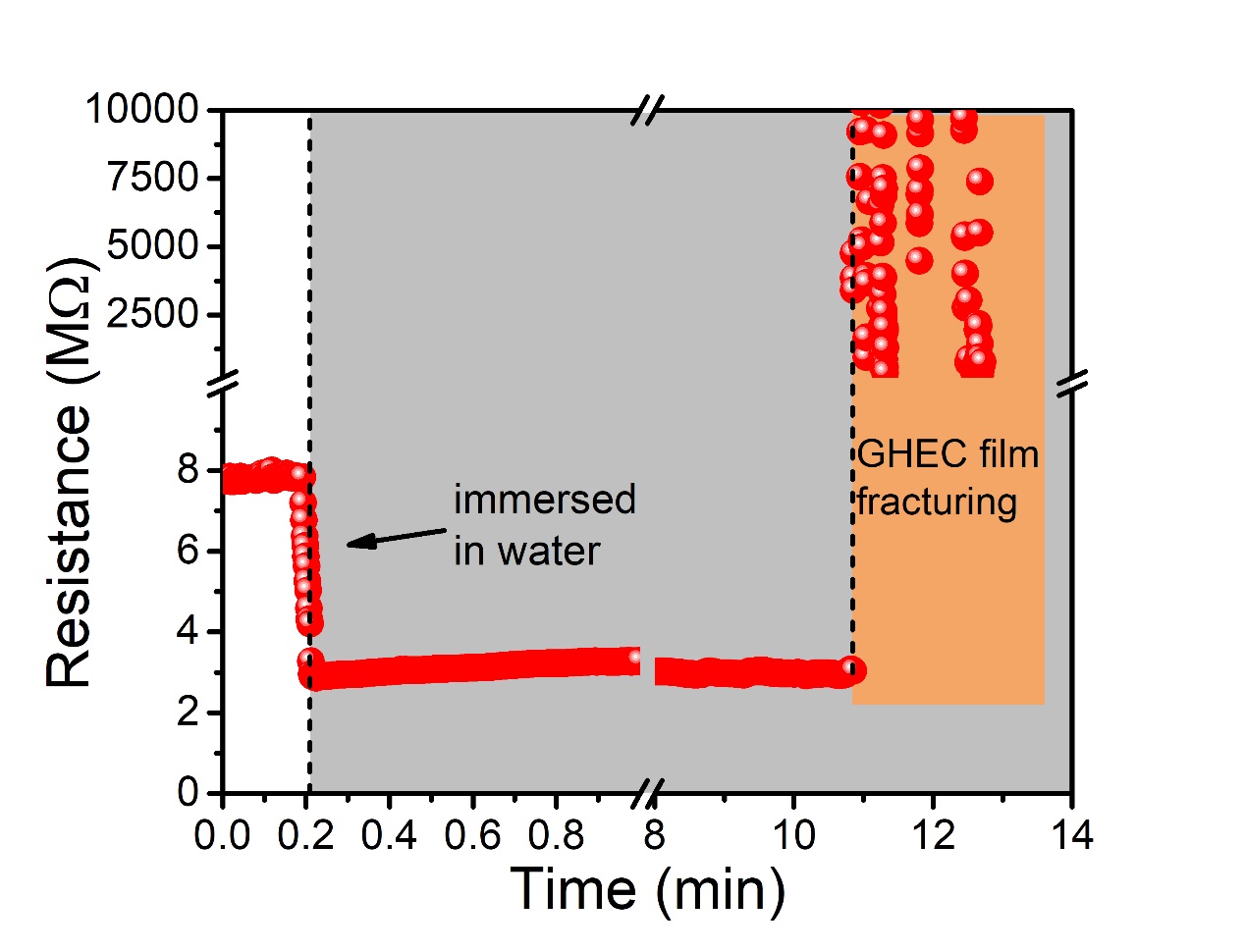


**Figure S8.** Resistance of GHEC (*m* = 20) gel film during degradation.


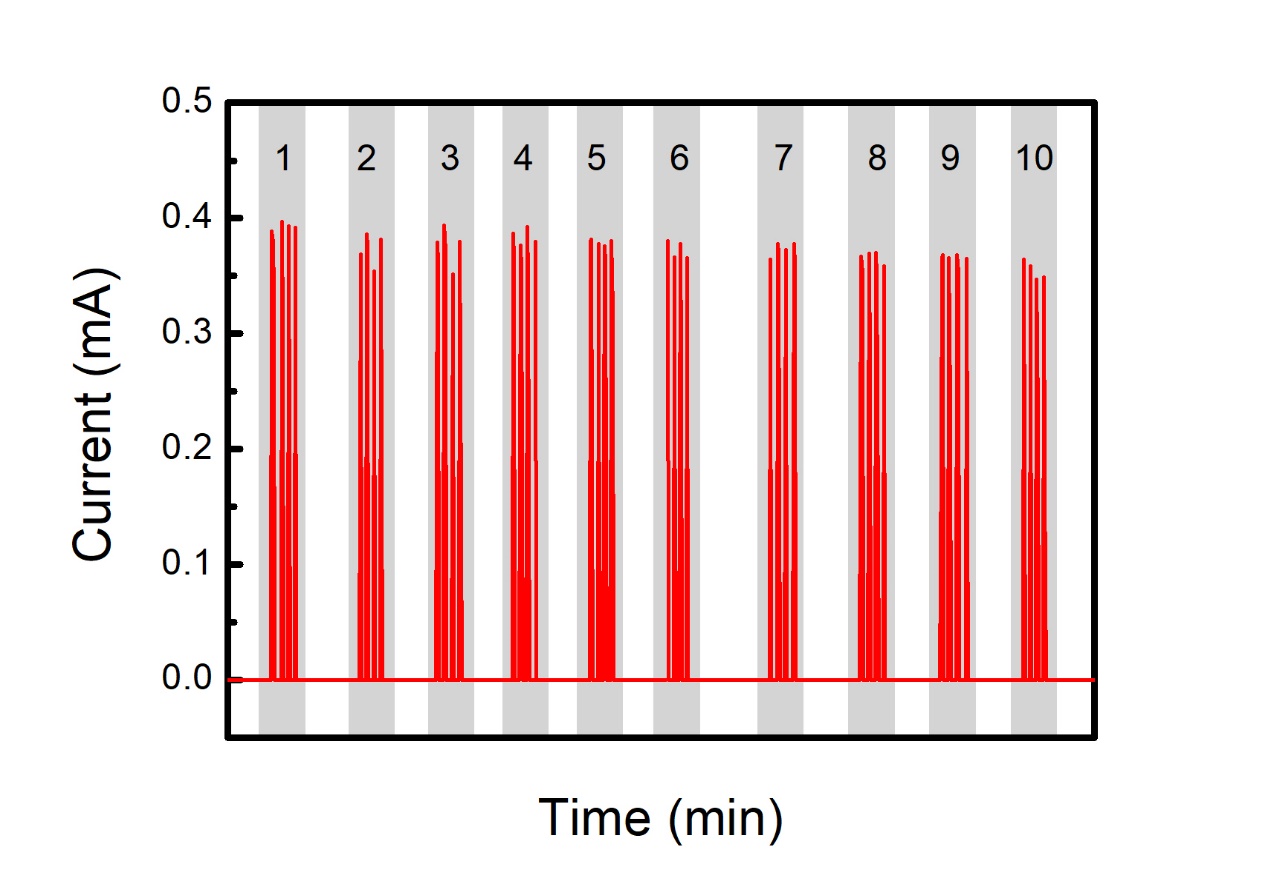


**Figure S9.** Real-time pressure detection of the electronic skin (fabricated by coating SWCNT on GHEC gel film) on the manipulator ring finger during 10 cycles failures and recoveries respectively.

**Supplementary Movie Captions.**

**Supplementary Movie S1. Mechanical healing efficiency test.** Five pieces of GHEC elastomeric gel films formed a cross-shaped film after adequate self-healing. The green GHEC elastomeric gel was fabricated by introducing green pigment into GHEC elastomeric gel, which will not affect the properties of GHEC elastomeric gel. Then the cross-shaped GHEC elastomeric gel film was stretched by a biaxial extensograph to test the mechanical healing efficiency.

**Supplementary Movie S2. Electrical property healing efficiency test.** A battery-powered circuit was constructed by connecting the LED, AgNW/GHEC elastomeric gel film, and power source in series to prove the potential of electromechanical self-healing for electronic circuits. The LED light in the series circuit are extinguished once cut off from the self-healing conductive elastomeric gel and then were lit again after transitory healing.

**Supplementary Movie S3. Real-time monitoring of self-healing, transient stretchable electronic skin.** The self-healing, transient stretchable electronic skin was fabricated by assembling a stress-sensitive layer of single wall carbon nanotubes (SWNTs) on a multifunctional GHEC elastomeric gel film to form an electronic skin. The electronic skin was assembled onto the curved surface of a manipulator ring finger by wrapping around the finger since the cross section of the electronic skin automatically heals in environmental conditions. Then, the real-time current of the electronic skin was measured using a digital source metre (Keithley 2602A) under a given voltage of 1 V. To simulate a real mechanical injury, a scalpel was used to cut a wound on the electronic skin and after a short period for self-healing, the manipulator ring finger regains its sensing ability. Finally, the electronic skin was removed and immersed in water to degrade.
